# Boundary spanning and identity work in the clinical research delivery workforce: a qualitative study of research nurses, midwives and allied health professionals in the National Health Service, United Kingdom

**DOI:** 10.1186/s12961-021-00722-0

**Published:** 2021-05-04

**Authors:** A. McNiven, M. Boulton, L. Locock, L. Hinton

**Affiliations:** 1 Nuffield Department of Primary Care Health Sciences, University of Oxford, Oxford, United Kingdom; 2 Department of Nursing, Oxford Brookes University, Oxford, United Kingdom; 3 Health Services Research Unit, University of Aberdeen, Aberdeen, United Kingdom; 4 The Healthcare Improvement Studies Institute, University of Cambridge, Cambridge, United Kingdom

**Keywords:** Research delivery, Professional identity, Research nursing, Qualitative research, Adjustment, Boundary spanning, Clinical research, United Kingdom

## Abstract

**Background:**

Research nurses, midwives and allied health professionals are members of an important emergent profession delivering clinical research and, in the United Kingdom, have been the focus of considerable investment by the National Institute for Health Research (NIHR). This paper considers the experiences of research nurses, midwives and allied health professionals in relation to professional identity work, recognizing these are coproduced alongside others that they interact with (including patients, clinical staff and other research staff).

**Methods:**

Semi-structured interviews were conducted with 45 nurses, midwives and allied health professionals in the UK about their experiences of working in research delivery. Interviews were transcribed verbatim and thematically coded and analysed.

**Results:**

Our analysis highlights how research nurses, midwives and allied health professionals adjust to new roles, shift their professional identities and undertake identity work using uniforms, name badges and job titles as they negotiate complex identities.

**Conclusions:**

Research nurses, midwives and allied health professionals experience considerable challenges as they enter and transition to a research delivery role, with implications for their sense of professional identities. A change in the work that they undertake and how they are (or perceive they are) viewed by others (including clinical non-research colleagues and patients) has implications for their sense of professional and individual identity. The tensions involved extend to their views on symbols of professional identity, such as uniforms, and as they seek to articulate and demonstrate the value of their conjoined role in *research* and as a *healthcare professional*, within the unfolding landscape of health research. We embed our study findings in the context of the newly emerging clinical research practitioner workforce, which further exacerbates and complicates the role and identity complexity for nurses, midwives and allied health professionals in research delivery.

## Introduction

Recent growth in clinical research activity—in the United Kingdom particularly through the National Institute for Health Research (NIHR)—has generated the new professional roles of research nurses, midwives and allied health professionals (R-NMAHPs) who now play an important role in delivering clinical research [9]. In this paper, we consider the identity work of R-NMAHPs as a relatively new professional cadre, in the context of their boundary-spanning roles requiring them to work across and between the boundaries of different groups. We explore how R-NMAHPs themselves navigate and articulate tensions in relation to their professional identities, how conceptualizations are influenced by others they interact with (including patients, clinical staff,^1^ and other research staff), and the work involved in forming and maintaining new professional identities.

## Background

### R-NMAHPs: an emergent profession

R-NMAHPs have been the focus of considerable interest, strategy and leadership investment by the NIHR (e.g. [18–23]). Clinical research nurses are described by the NIHR as “crucial to delivery research” [24], whilst research allied health professionals (AHPs), including art therapists, dietitians, paramedics, physiotherapists, radiographers, speech and language therapists and others, are acknowledged as “play[ing] a vital role in the delivery of high quality, patient centred clinical research across the NIHR” [25]. The diversity of activity and the boundary-spanning nature of these roles is acknowledged; they “provide and deliver high quality patient care, deal with data collection, follow-ups, patient groups and industry” and “also develop and build multidisciplinary teams that deliver research” [24].

There is a growing literature on the clinical research delivery workforce in the United Kingdom, with the majority focused on nurses (e.g. [3, 4, 28, 30]. Research midwives are sometimes grouped alongside research nurses, whilst there has been relatively little published literature specifically on the experiences of research AHPs. R-NMAHP roles typically focus on “delivering research”, including activities such as participant recruitment (for example, identifying and approaching eligible patients, taking consent), data collection (for example, collecting patient data and biological samples), delivering interventions, and data entry and management, although there is considerable diversity across sites, teams and studies. R-NMAHPs are largely excluded from the development, analysis and write-up stages of research, with these activities usually undertaken by principal/chief investigators and clinical academics (including those who are independently undertaking research, including clinical but also health and social science in nature, as NMHAP researchers). The roles draw on multiple skill sets, bringing together existing clinical expertise alongside new research skills. Their work straddles the worlds of clinical practice and research, requiring skills from both while not rooted entirely in either.

The research delivery workforce is heterogeneous. In their study, Boulton and Hopewell [4] report almost a quarter of staff delivering clinical research were from backgrounds other than nursing. These included pharmacists, physiotherapists, psychologists and radiographers as well as healthcare assistants, assistant practitioners and data managers. The reasons for this diversity are unclear but could reflect both difficulties in recruitment to posts as well as a recognition that, for some studies, individuals with different backgrounds may bring more appropriate skills.

### Clinical professions and boundary work

The development of new professional roles raises the issue of professional boundaries, boundary work and boundary spanning [10, 12, 14]. Gieryn [12] articulated the notion of boundary work in analysing the “struggle for authority” (p. 784) between scientists and non-scientists, and there is an extensive social science literature dedicated to understanding clinical professions, power and knowledge [7]. Grant and Guthrie [14] explore the medical and administrative boundary work in primary care and, drawing on Fournier [10] and Gieryn [12], refer to boundary work as “the process whereby boundaries or divisions between the fields of knowledge of particular professions are created, challenged or reinforced” (p. 44). The strategies employed to do this professional boundary work include codifying knowledge through formal curricula or practice guidelines, restricting admission to the profession (for example, by examination), and excluding the amateur or unqualified to maintain power and prestige [16].

There is now a wide literature on boundaries both between and within healthcare professions. Allen [2] and Powell and Davies [27] examined how hierarchical boundaries between nursing and medicine are sites of tension and blurring, as tasks are renegotiated, delegated, tacitly adopted or usurped. But boundaries may exist and shift within professions too. Freidson [11] argued that whilst medicine retained a high degree of collective autonomy, it became internally stratified as a strategy to defend against external control. Whether this was successful or led to further weakening of medical autonomy and power is still debated [5].

Nasir et al. [19] describe boundary spanning as “the role of individuals who work in groups but who have ties across boundaries that divide their colleagues” (p. 5). In the organizational studies literature, the term originated from observing the need for organizations to find ways of sharing information and insights across highly specialized knowledge silos to maximize innovation (e.g. [31]). Whilst boundary-spanning roles are assumed to be of positive organizational value, Crosno et al. [6] note that individuals occupying such positions often experience a high degree of role stress. This results from both role conflict (conflicting expectations or demands from different stakeholders in the person’s network) and role ambiguity (uncertainty about what an individual should do to fulfil their responsibilities).

### (Nursing) identity, identity work and invisible work

Whilst the topic of professionalism features widely in midwifery and individual allied health professions literatures, it is particularly prominent in the nursing literature. Strategies to break free from “handmaiden” status for nurses have included progressing to a degree-led profession and development of new advanced roles (e.g. clinical nurse specialist, nurse consultant). Uniforms are emblematic of these professionalizing strategies, which include abandoning frilly caps and starched aprons, introducing trousers, and sometimes having no uniform at all. As Timmons and East [29] argue, “uniforms are the most visible symbolic manifestation of a professional of occupational group” and “a way of delineating professional boundaries and demonstrating occupational hierarchies”.

Allen [1] argues that, in addition to the centrality of care and caregiving in constructing a sense of identity and purpose, nurses are also crucially engaged in “organizing work”. This important organizational knowledge and action, which can be summarized as “care trajectory management”, is largely “invisible work” which can be regarded as taking nurses away from the “real” work of nursing.

Foregrounding caregiving as the defining feature of the nursing profession also neglects the diversity of nursing roles, and this also holds relevance to midwifery and allied health professions. In this paper, we add to this diversity the heterogeneous roles of the R-NMAHPs whose daily activity may consist almost entirely of (invisible) organizing work. Whilst the themes of invisible work, professional boundaries and identity have been explored largely in the nursing literature, we suggest the concepts are relevant to a wider range of healthcare professional (HCP) roles involved in research delivery.

## Methods

A qualitative interview study explored the experiences of a sample of R-NMAHPs in the United Kingdom. Forty-five in-depth interviews were conducted with individuals currently or previously employed as research nurses, midwives and AHPs. The study was funded by the NIHR Oxford Biomedical Research Centre (BRC) and approved by the Berkshire NRES Committee South Central.

A maximum variation sample was sought [8], which included duration of experience in the role, clinical areas, and work settings and/or employers. Participants were recruited through a variety of sources, including contacts at primary and secondary care settings, Clinical Research Network mailing lists, social media and snowballing. All potential participants were given detailed information sheets and opportunities to discuss involvement. Participants could choose whether to be video- as well as audio-recorded for interview extracts to be published on Healthtalk.org [15] in video, audio and/or written formats. All transcripts were transcribed verbatim for analysis.

The interviews were conducted by AM between December 2017 and July 2018, in participants’ homes or alternative meeting spaces. Interviews were semi-structured, with an opening question inviting participants to talk about their experiences as a research nurse, midwife or AHP and with follow-up prompts (e.g. on participants’ routes into research, what helps or hinders their research activities, their views on the profile of research in their profession, and their relationships with colleagues).

A coding framework was developed by AM based on interview content and relevant literature, and refined throughout coding in discussion with LH. NVivo12 software was used to organize the data and facilitate the coding process using thematic analysis. Coding reports were shared with co-authors and analysed thematically.

## Results

Forty-five in-depth interviews were conducted with individuals currently or previously employed as research nurses, midwives or AHPs (see Table 1 for details).

**Table 1:** Participant backgrounds and sample

Participant details	Sample (*n* = 45 total)
Research nurse	24
Research midwife	9
Research AHPs Physiotherapist Radiotherapist Speech and language therapist Art therapist Health visitor Paramedic Podiatrist	12 4 2 2 1 1 1 1
Less than 2 years in role	10
3–5 years in role	14
6–10 years in role	15
11–15 years in role	2
16 + years in role	4
Female	37
Male	8
White British	33
White European (Italian, Romanian, Portuguese)	4
Indian	2
British Black African	2
British Pakistani	1
Mixed	1
White American	1
White Canadian	1

Experience in the role ranged from a few weeks to more than 25 years. The clinical areas included oncology, paediatrics, reproductive health, sexual health, metabolic and endocrine health, mental health, end-of-life, critical care and emergency medicine, diabetes, musculoskeletal, dermatology, cardiovascular, neurology and gastroenterology. The work settings and employer contexts included large teaching hospitals, district general hospitals, primary care, palliative care, research networks, universities and commercial organizations. Some participants held more than one post, for example two or more contracts for research posts with different employers, and/or separate clinical non-research posts (including bank shifts). A few identified as nurse researchers, midwife researchers or AHP researchers leading their own research, including those undertaking or who had completed academic research qualifications such as a PhD; these individuals had previously worked in, or continued to also work in, research delivery roles. The distinction between the roles was sometimes a source of confusion and led to some R-NMAHPs feeling disappointed that they were not or could not be involved in other stages of research. Two participants were qualified in both nursing and midwifery, and one participant was qualified in both midwifery and health visiting.

A diverse range of experiences of, and views on, professional identity featured in the interviews. We identified three prominent themes: (i) adjustment to new roles; (ii) shifting identity; and (iii) complex identities and identity work (including uniforms, name badges and job titles as symbols of complex identity).

### Adjustment to new roles

Many R-NMAHPs found adjusting to a new role in research delivery challenging. Some felt they had a good grounding in research activities before starting their first post, but others arrived with little knowledge or practical experience. R-NMAHPs described having to adjust to working with new colleagues or working with existing colleagues in new ways, adopting different patterns and paces of work and undertaking new types of activities or approaching existing activities and responsibilities in a different way. The realities of the role could be very different from expectations.

*I didn’t realize how much admin there was going to be. And how much physical desk work and paper work. […] At the very beginning I just thought “this is never ever going to sink in”.* (R-N01)^2^

*There is a little bit of a dance and a re-adjustment. […] I won't say that it's disappointment but it's a regrouping of how perhaps I, or other people I can see, think of being a research midwife, because sometimes it's a little bit removed from the ideas.* (R-M01)

Some research midwives suggested independent working was less of an adjustment when they moved into research delivery, in contrast to other professions typically engaged in team-based clinical work:

*You're still doing the work on your own, you're still one-to-one with a woman, whatever it be—research or clinical. Because as midwives, they’re autonomous practitioners.* (R-M07)

Inconsistent approaches to, or the absence of, formal and informal training for new R-NMAHPs could contribute to feeling overwhelmed and disorientated. R-N02 had worked in research for 7 years and recalled, “I had to find out things for myself.”

*Back in 1991, we were just thrown into it.* (R-N04)

*I went from working my shifts to Monday morning starting in research and “right, here you go”. And sort of learnt a lot as I was going.* (R-AHP01 [paramedic])

In contrast, R-NMAHPs who had moved into the role more recently were more likely to have received more extensive and organized inductions, training and mentoring.

At first, clinical research often felt like a “different world” to that of clinical practice. Some adjusted quickly, but most found that it took several weeks, months or even years to feel more comfortable and confident in a research delivery post. During this time, some R-NMAHPs contemplated leaving the post and returning to a more familiar clinical (non-research) role.

*People said, “Are you enjoying it?”, and I thought ‘actually, do you know what, I don't know’. Because I don't know whether I'm doing it right, I don't know whether I actually know what I'm doing. I didn't. I think it took 12 months. […] I just had to ride it out.* (R-N06)

Having good relationships with others, including patients and non-research clinical staff, was an important component to this adjustment.

*[A colleague said] “You need to take some time to realize that you’re not a clinical physio anymore. That’s not what you’re employed to do. You’re a research physio and your job is to carry out the research to the best of your ability.” And sometimes that involves, for example, having a straight face, not engaging in conversation while somebody is carrying out a test. Not giving feedback on the results because it might skew the data in some way.* (R-AHP02 [physiotherapist])

*So when you go into a clinical setting as a research nurse, obviously your primary role is to, as you say, collect data for the study, recruit participants for the study. But of course you go in there with a nurse perspective. So a lot of what you have to do is perhaps adapt to a different role. And that's the sort of greatest challenge, I think, really. Understanding that you're not part of the clinical team.* (R-N07)

R-NMAHPs often spoke about aspects of their role which had endured, as well as what had changed. Continued patient contact and good communication skills were often highlighted as important, with the role of the R-NMAHP vital in bridging between the documented aims of the research (as articulated in patient information sheets, for example) and actual patient understanding.

*Some of the skills that you use in midwifery, you really, really need [… Like] communication skills.* (R-M02)

*The patient information leaflets come from the trials. But it’s your own communication skills it comes with. I’ve been nursing for 15 years now, it’s something you learn to do, and you work off their cues, their verbal cues, their nonverbal cues. And you talk in a language that they can understand. […] So, it’s all about the conversation that you’re having with the patient.* (R-N01)

Various clinical skills were highlighted, enabling research to happen more quickly and without relying on other clinical (non-research) staff to help.

*A lot of transferable skills came over with me that I didn’t realize would be as beneficial until you actually have to do it. […] If you need to take some bloods, you need to take them there and then.* (R-N01)

Some R-NMAHPs expressed concerns about de-skilling through a research role. Those who held two or more posts, such as a second job which was clinical (non-research) or undertaking bank shifts, suggested this could help maintain clinical skills. However, for some, it remained challenging because of rapid changes in healthcare technologies and practices in use. The sense that they were losing their clinical skills, or that others thought this about them, could be distressing and contribute to feeling that they were no longer a “proper” nurse, midwife or AHP.

Others rejected this notion of de-skilling. Instead, they saw these skills as becoming dormant or their role as evolving as new skills were acquired.

*We don’t de-skill, you learn a new set of skills. […] It’s just developing more and I don’t believe that those skills that you had there already are lost, you build on them*. (R-N02)

*You’ve still got all those nursing skills [like interacting with patients], we’re just probably utilizing them in a different way.* (R-N04)

*I’m a physio with extra skills […] rather than no longer a physio, who can do less.* (R-AHP04)

### Shifting identity

Many R-NMAHPS were drawn to research roles in search of a “change”, “challenge” and something “new” or “different” to do. Some moved into a research role seeking more family-friendly work patterns, highlighting an intersection of both professional identities and personal parental/carer identities in the career move. For some, becoming a research nurse, midwife or AHP meant moving into a new speciality at the same time as adopting a new role. Others were keen to stay in (or return to) a clinical area they had worked in before but to try a different role and develop new skills.

*I was ready for something new. […] [It] was just a natural progression for me and I think that obviously there are many transferable skills.* (R-N09)

Their own or others’ perceptions of a changed professional identity was sometimes experienced as a loss or threat. This was often articulated as feeling no longer a “proper” nurse, midwife or AHP. These concerns sometimes faded, but other times they endured and gave rise to considerable anxiety for some participants. This threat to professional identity sometimes stemmed from comments or actions from clinical (non-research) colleagues and patients/participants.

*I can remember people saying, “Oh, didn't you use to be a nurse”, and things like that to me, when I met them, still on the ward, but not dressed in my uniform. So yes, it does have an identity impact, definitely. […] For some people, maybe from being young, they've always wanted to be a nurse when they grow up, to then suddenly be offered an opportunity to not look like a stereotypical nurse, or not be on a ward in a clinical area, might then make them think 'yes, well I have lost a bit of identity'.* (R-N10)

*A lot of people just thought ‘well I could do that, sitting in an office at a desk’, so that was their interpretation of what a research midwife would be. There was quite a bit of negativity.* (R-M04)

*We still do face that ‘we don’t have patient contact, we’re not proper nurses’.* (R-N04)

Sometimes R-NMAHPs had heard or seen challenges to professional identity made about others which they then internalized.

*One of the managers there said to me, “Oh, so somebody who had come and done their placement with us was training to be a speech therapist, then went on and did research, and I thought, oh, what’s the point; we’ve completely wasted all of our efforts doing clinical training with this person, ‘cause she’s not going to be a clinician.” And that really stuck with me.* (R-AHP03 [speech and language therapist])

Pre-empting difficult exchanges, some developed strategies such as using a “stock response” when asked about their job.

*If somebody says to you, “What do you do?” I now say, “I’m a nurse by background.” That’s how I’ve started to address it, because I recognize I don’t function in the typical view of what a nurse does. And partly that makes me a little sad sometimes, ‘cause I think I always loved being a nurse and I am a nurse, in my heart I’m a nurse. But actually, that’s not what I do on a day-to-day basis now.* (R-N11)

A move into research could prompt people to confront and re-evaluate their own assumptions and judgements about R-NMAHPs and their activities.

*I kind of thought the research nurse job was, to be honest, a job that somebody in their late 40s, early 50s, who’s worked a hard career and wants a bit of an easier job, that was my honest perception. Now that I’ve worked in that area, and particularly in the early phase clinical trials, I know that is not the case at all and it actually is quite difficult work.* (R-N12)

Emphasizing the ways in which they continued to “work” as a nurse, midwife or AHP and use their expertise could help mitigate a sense of losing their professional identity. This included using their clinical skills and good patient communication.

*My background in nursing has helped, for the language mainly. I understand when they’re talking about tumours and things like that, but it’s been a massive learning curve. It’s been really, really interesting and I still love it, and I’m so glad that I made the change.* (R-N01)

*If a patient says to me, “Oh, I haven’t had a cup of tea,” or whatever, then I’ll go and get one rather than ask somebody else to go and get one or if they want to go to the loo [….] Just because you’re not working there clinically doesn’t mean that you can’t help out a little bit.* (R-N5)

The way that R-NMAHPs perceived individuals as patients or participants also highlighted a complexity in their own co-constructed sense of professional identity.

*Researchers use the word “participants”, and they are participating but that doesn’t stop them being our patients. […] I still tend to call them patients. […] I think that might be the nurse in me, maybe. […] They’re patients first, participants second.* (R-N13)

Spanning the boundaries of their role, however, could also lead to some confusion about its limits. The blurriness of the boundaries could present difficulties for R-NMAHPs but also for patients/participants and for (non-research) clinical staff. Recognizing the differences in roles and learning how to negotiate them in their research post sometimes required un-learning habits and practices they might easily “slip into”. This was further complicated by some R-NMAHPS holding two or more posts and so might see the same individuals when in their clinical (non-research) role and in their research NMAHP role.

Whilst some experienced a threat to or loss of their professional identity, others felt the move into research had enhanced it. They brought skills, knowledge and values from their previous experiences into their research roles and continued to use them on a frequent basis to deliver research *alongside* patient care.

*I am a nurse, and my job is to make sure that my patients are well supported, well cared for, that I have considered their whole life, given holistic care. And it just so happens that a clinical trial is the vehicle to do that. […] So I’m the nurse, not the research nurse.* (R-N12)

Some research midwives and AHPs emphasized it was not only nurses who could work in research delivery roles and saw themselves playing a role in diversifying the workforce.

*It is so nursing-biased, that you do feel like you’re blazing a trail for another profession because you are, you have to fight a little bit for recognition.* (R-AHP02 [physiotherapist])

A move into research delivery meant R-NMAHPs also positioning themselves in relation to new individuals, groups and organizations of the research infrastructure, including study principal investigators (PIs), chief investigators (CIs), clinical academics and study site coordinators. R-NMAHPs' relationships and communications with these research colleagues varied—depending on their work settings, employment context and the studies they worked on—with continuums of close to distant relationships, and frequent to rare communication. Working with research colleagues could highlight career progression and different pathways to R-NMAHPs, including independent researcher roles. However, research colleagues could also be felt as another group to which R-NMAHPs felt uncertainty and ambiguity about belonging. Some R-NMAHPs described situating themselves as working alongside and between, rather than necessarily within, these different groupings.

*I remember clearly thinking, “I don’t know anyone, I don’t know who these people are.” I remember meeting the trial coordinators, PhD students, the PI and the CI, and I remember thinking, “I just don’t know where to place these people.”* (R-M08)

Some R-NMAHPs felt that their professional healthcare backgrounds were viewed dismissively by academic and other research colleagues, which hampered their working relationships. R-N02, who led a team of research nurses, explained:

*I found that when you go and you discuss at the higher level with academics and staff, […] some of them they seem to be less receptive of what you’re saying after you mention the “nurse” [part of your background]. I’ve even been advised to ditch the “nurse manager” from my title and just to be a “research manager”, just to be able to talk to a variety of people.* (R-N02)

R-N02 suggested that perceptions of the nursing profession and behaviours such as downplaying achievements were interwoven with issues of gender, and that this dynamic could also affect working relationship for research nurses with other research colleagues:

*You will meet so many quiet nurses in this hospital and you wouldn’t know anything about their achievements because we are not trained to do that, and probably has to do with both the job [nursing] and the gender. We don’t go out and shout loud what we’re doing and what we can do and what we’ve achieved.* (R-N02)

### Complex identities and identity work: uniforms, name badges and job titles

Those who had typically worn uniforms as clinicians (such as hospital-based nurses and midwives) often saw wearing a uniform in their research role as helping maintain their professional identity and status in the eyes of clinical colleagues and patients. Uniforms could facilitate rapport and trust by communicating that R-NMAHPs are knowledgeable about clinical and healthcare.

*I want to make sure that I'm properly integrated within their [clinical] team, so they don’t just see me as a research midwife but I'm actually a midwife. And they’ve tried to put me in a uniform as well to help with that.* (R-M03)

Although continuing to wear a uniform helped some R-NMAHPs feel a sense of continuity with their clinical background, it could also lead to confusion.

*When I started, I was told it was a non-uniform job. But I could wear a uniform if I wanted. So because I was struggling with not being [laugh] a nurse any more, I did wear my nurse's uniform for a very short while. But obviously became mistaken for a ward nurse the whole time. So I went into non-uniform pretty quickly.* (R-N14)

Some felt it was important to distinguish themselves from clinical staff by *not* wearing uniforms.

*The reason that we don’t wear a uniform is that we are seen as separate to clinical work and clinical staff, and I think that does help […] There's always that thing about is a uniform a barrier, or is it an identifying professional type of outfit; I don’t know.* (R-N15)

*When I put my uniform on, I feel more of a sense of responsibility to the unit and I feel like I could be called on at any time. So it’s almost a conscious choice sometimes to not wear a uniform, and therefore, can’t be called upon to do anything. That sounds terrible, doesn’t it? ‘Cause I do value what they’re doing, and I have my uniform in the drawer ready to go, if it was needed.* (R-N11)

For those who held a clinical (non-research) post or took bank shifts in addition to their research post, the use of uniforms could help set the boundaries and expectations of each role to patients and clinical (non-research) staff whom they saw in both capacities.

*A day where they’re working full time research and then the next day they’re working clinically, or it might be that they work a Wednesday morning in the research office but then lunchtime they change into their uniform and they are working clinically. So I make it quite clear when you are doing which role.* (R-AHP05 [physiotherapist])

*[Uniforms] does help with our team because actually sometimes they encounter families in both capacities, both as a bedside nurse and as a research nurse. […] We have to be very careful that we don’t make them feel coerced because you’ve suddenly gone in with a different ‘hat’ on.* (R-N11)

The absence of a uniform (such as tunics/scrubs) could differentiate those in research from clinical (non-research) staff, whilst wearing an identity badge could still affirm their professional affiliation.

*I don’t wear a uniform. No, there doesn’t seem to be any confusion. But I’m still a midwife […] It says on my badge and they [patients] can still ask me questions.* (R-M04)

However, others felt that an identity badge was too subtle or insufficient for the complex task of communicating professional identity to themselves and others.

*I don’t really like the idea of a uniform in general. But I think it makes people identify with who you are straightaway. ‘Cause if I’m just dressed like this and I walk up to someone, and maybe you’re not listening or, and I say “I’m—”, they might miss that I’m a midwife, and they can see my badge, but they think ‘who is this person?’* (R-M05)

For those R-NMAHPs who wore uniforms, or expressed the wish to do so, there were different views on how the style/colour of a uniform could denote their HCP backgrounds *and* differentiate them as working in research. A research-specific uniform, or multiple uniforms relating to different grades and roles within research, could visually highlight the presence of research in the clinical environment and help normalize it for clinical (non-research) staff and patients.

*I know in some places research staff don’t wear uniforms but I feel quite strongly that we do wear uniforms because I think that I’m a nurse and in fact in the past we had lots of discussions with my manager at the time about perhaps we all just wear the same uniform and it’s a specific research uniform. And I’m a nurse and I’ve trained to be a nurse, I’m a Sister, and I don’t want that taken away from me because that’s how I identify myself and that’s what I want to be. […] Now we’ve got the CNS [clinical nurse specialist] uniforms [in my team], the doctors know that we’re not part of the ward team […] so that’s actually helped quite a bit. But I do feel quite strongly about uniforms because I think it identifies us*. (R-N04)

*And our uniform is different. And they will ask, “Oh, what's this uniform, what do you do?” […] Because there are, because our colour is grey. So now there are lots of grey uniforms you can see around.* (R-N08)

*I think we're in uniform more so for the cohesion, so people can actually look at us and be like, “Oh, they're part of the research team” [at our Trust] as opposed to anything else.* (R-M03)

Some AHPs suggested that uniforms were less important to their sense of professional identity compared to nurses.

*It’s the psyche of nursing and ward work and hierarchy, and I can see why that works on a ward. In a ward environment, you need to know who’s doing what. […] Because the people who are in research have come from that clinical background, it feels like that’s all that they know, so they replicate that in their own behaviour.* (R-AHP02 [physiotherapist])

Once working in research, however, and particularly in environments where research nurses were the main research delivery workforce, uniforms could become more salient to R-AHPs. There were different views on whether the same uniform should be worn by all R-NMAHPs in a team/organization. A research-specific uniform could signal a shared identity in research delivery, but for some R-AHPs, it masked different professional backgrounds that they felt mattered.

*We all wear the same uniform, so irrelevant of profession, we all wear grey tunics in research [at our Trust]. And I think it's about trying to sort of, for me, reach the rest of the Trust and say, “Actually, grey doesn't mean nursing, grey just means research, and actually all of us can contribute in this way.”* (R-AHP04 [physiotherapist])

*When I started, I had a conversation with somebody quite high up, who asked for my opinion about what allied health professionals should wear, because they had a set colour with a set trim for different levels within research nursing, and it worked for the nurses, but if you go in as something other than a nurse what do they put you in? I gave an opinion, I was told that they were thinking about it, it’s now nearly two and a half years down the line, and nothing has happened. And I am currently wearing the uniform of a) a nurse and b) a band lower than I am, because they don’t know what to put me in. […] So, I’m in an environment where there’s not a lot of people like me, and I’m dressed in something that is me masquerading as something else.* (R-AHP02 [physiotherapist])

In settings where the research delivery workforce included those who were not NMAHPs by background, the question of uniforms raised additional uncertainties. R-N14 wore a clinical nurse specialist uniform and felt “it means something to patients”, but her non-NMAHP research colleagues did not wear a uniform of any type.

*The clinical trials officers [CTOs] aren't allowed to wear a uniform because they're not a nurse. And that's difficult. They want to wear a uniform and I can see why. And they're going out and doing exactly the same job as me. And it's just as clinical. So personally, I think it would be nice if we all had the same uniform […] some sort of shared identity with the CTOs*. (R-N14)

An added complexity concerned job titles. Although R-N14 self-identified as a research nurse, she was technically employed in her current post as a “clinical trials officer”, with this title giving no explicit mention of her nursing expertise—and despite her uniform denoting it. The omission of an individual’s professional identity in a job title was sometimes described as having practical advantages (for example, drawing boundaries around responsibilities) but disadvantages in terms of camaraderie with clinical colleagues:

*I've heard of [employers of research midwives] calling them “associates” and then they've [laugh] got “(midwife)” in brackets. Which I just think is a really bizarre thing to do. I'm not sure if it's helpful for them. It might be helpful for them in situations like that [where clinical colleagues might ask a research midwife to help with patients], but you are still a midwife, you're just a different kind of midwife.* (R-M05)

Other times, a job title specified a different professional background to that of the actual post-holder. R-M06 was a midwife and R-AHP02 a physiotherapist, but both were officially employed as research *nurses*, reflecting assumptions about the typical background of the research delivery workforce:

*I looked for those [research nurse/sister job advertisements] and looked at the person spec and what they were asking for, I couldn’t see any reason why as a physio I would be any less able to do that job than a nurse. I mean I think sometimes it can be quite study dependent.* (R-AHP02 [physiotherapist])

There were many variations of job titles for those employed in research delivery. R-N03 named six she knew of within her organization and added that there are “many more job titles across the country”. She also saw the breadth and ambiguity around job titles as a problem:

*I believe it really poses a problem to the [research nurse] workforce having such a huge variety. It is difficult enough to demonstrate what we do, to raise our profile and show our worth—having a multitude of titles does not show how valued the research nurse role really is. […] We all know the value but without evidence our roles are likely to be (and in some cases already are being) swapped for lower levels, grades and those without professional qualification.* (R-N03)

## Discussion

The research nurse role is relatively well-established when compared with the more recent development and growth of similar roles in, for example, the allied health professions. Nonetheless, research delivery roles of NMAHPs are emergent, and their shaping is ongoing. The ways that R-NMAHPs articulate their professional identities (and have their professional identities articulated for them) relates to and draws upon the wider context and groups of people whom they work with, for or amongst. This includes, but is not limited to, other R-NMAHPs, clinical colleagues, research colleagues, patients and the public. The sense of alienation and rejection from their clinical colleagues that R-NMAHPs sometimes describe, and their own internalized concerns about this, highlights how undertaking research delivery roles is still seen as a move away from or out of the caring profession—despite attempts to change this perception. Patients/participants can inadvertently reinforce these messages too.

Our study builds upon the call by Tinkler et al*.* [30] to further explore the professional identities of research nurses, and we expand the remit to consider the experiences of research midwives and research AHPs. Tinkler et al*.* ([30], p. 8) describe how clinical research nurses “appear to struggle with reconciling the dichotomy between clinical practice and experimental science, describing themselves as nurses first and foremost, separating themselves from nonclinical recruiters to maintain their professional identity as a nurse”. Our findings resonate with this description and frame it as a struggle for research nurses, midwives and AHPs to absorb boundary-spanning activity into their new/revised professional identities. We suggest that because boundary spanning is embedded in the remit of the “research delivery” role, it could be embraced by R-NMAHPs as an asset in their articulations of professional identity, but note that instead it tends to underpin concerns about *not being a “proper”* NMAHP, nor researcher. At the same time as feeling that clinical colleagues and patients do not always understand and value their research delivery activities, a further challenge to the ongoing establishment and progress of the R-NMAHP workforce comes from initiatives to professionalize non-HCPs to deliver research. In the United Kingdom, clinical research practitioners (CRPs) have been identified as a new cadre involved in multiple stages of the research process [9]. The recent establishment by the NIHR of the CRP Directory represents efforts to define, identify and accredit a group within research delivery that exists alongside, but is not the same as, research nursing; however, this approach may be shifting.^3^ Whilst the role of CRPs chimes with that of R-NMAHPs in terms of “working in a research delivery role that involves direct contact with patients and activities in clinical environments or other health and social care research settings”, a differentiation can be made between professional backgrounds, as CRPs are “not registered with a healthcare profession” [26]. With our interviews completed between December 2017 and July 2018, predating the development of the CRP Directory for example, the consequences of this emerging and professionalizing CRP workforce for the R-NMAHPs in our study were not always explicit. However, there are glimmers of recognition of CRPs as an emerging threat or complication to the ongoing establishment and progression of the R-NMAHP workforce within our data. This can be seen in concerns expressed about those without professional healthcare qualifications or with only lower levels of qualifications displacing them in the research delivery workforce. The sense of inappropriateness expressed by some of our participants resonates with previous literature exploring, for example, research nurse manager views on non-nurses performing clinical research activities [17]. As demonstrated in our findings, tensions around R-NMAHP and non-NMAHP (e.g. CRP) roles can materialize in both views about and experiences of the uniforms worn, or not worn, by those delivering clinical research.

The approach adopted in the United Kingdom of developing and professionalizing CRPs to expand health research contrasts with the approach being undertaken elsewhere. In a United States study, Jones et al. [17] highlight the importance of identifying boundaries between the roles of nurses and non-nurses in order to mitigate risks related to study participants and the validity of the study: “The FDA [Food and Drug Administration] identified a need for a broader binding policy in defining the delegation of research activities to clinical research personnel, thus requiring those activities only to be delegated to nurses.” By contrast, in the United Kingdom, driven by pressure to increase the number of studies undertaken, particularly commercial/industry studies which provide substantial income to the National Health Service (NHS), the NIHR has looked to recruit from a wider pool of individuals without professional qualifications in the healthcare field. While this may address the immediate need for a larger research delivery workforce, it may also lead to tensions between those who are experienced HCPs and those in similar research delivery roles who are not. As budgets are squeezed, CRPs may be seen as more “cost-effective”, and opportunities for R-NMAHPs may decline. This shift towards recruitment of CRPs is evident in the efforts within the NIHR to seek accreditation by the Professional Standards Authority for a CRP register and to “lay the foundation for defining professional identity and establishing an accredited register for this diverse group” [26]. Questions remain, however, over the safety of patients and study participants when those who are caring for them have limited clinical skills.

The question of what added value comes from a research delivery worker having professional NMAHP qualifications is a complex one, grappled with in different ways by the R-NMAHPs in our study. Efforts to demonstrate why it is important that health research takes place *within* and *alongside* the context of patient care, and why R-NMAHPs are most suited to facilitate this, are ongoing. As R-N03 described, it is both well known (“*we all know the value*”) and difficult to demonstrate (“*without evidence*”). Some elements of the value are challenging to calculate—for example, R-NMAHPs often report that patients/participants ask questions or request advice during research encounters that, as qualified and experienced HCPs, they are able to respond to appropriately. The value of this is not only how *often* these opportunities to further support patient/participant health occur, but also whether research opens up additional or more timely opportunities for such topics/questions to be raised that might not be aired with (non-research) HCPs, and what benefit this gives.

As our data show, the question of whether it is important that those delivering research are NMAHPs is complicated by the variety of views R-NMAHPs themselves hold. Many felt it was important that the individuals delivering research to patients/participants have HCP backgrounds, and that their roles appropriately integrate elements of their *caring* with their *research* abilities, whilst others questioned whether professional backgrounds were necessary. These uncertainties may reflect the realities of some study activities where NMAHP expertise is less significant and referral to clinical colleagues if patients/participants have queries may be sufficient. Yet it may also reflect the chronic undervaluing of their work that many R-NMAHPs have experienced, especially those who have worked in the role for many years. Whilst some study participants expressed uncertainty over the value of their nursing, midwifery and AHP backgrounds to their research roles, all participants demonstrated ways in which their backgrounds had been an asset that was not available to non-HCP equivalents.

The experiences and views of patients/participants about R-NMAHPs facilitating their research participation have not been explored in detail to date. This presents another avenue through which it might be possible to elicit whether, and how, the professional backgrounds of research delivery staff matter, and to compare these insights with views on non-HCP research delivery staff. As described, some R-NMAHPs recognized that there is a lack of public understanding about their roles and that what the role entails does not align with the public image of NMAHPs. This may mean that patients/participants are not confident or aware of the benefits derived from R-NMAHP knowledge and skills. Raising the profile of R-NMAHPs to the wider public and to patients in general could not only expand understandings of modern NMAHP careers but also increase recruitment to studies and gains from the added value that those with NMAHP backgrounds can provide. This is timely, as R-NMAHPs around the world contribute to the prevention of disease and promotion of health—for example by recruiting patients to clinical trials and providing support throughout the trial, contributing to the development and testing of new drugs including the new vaccines for COVID-19 and, more broadly, delivering the interventions that will ultimately improve patient care and population health.

## Strengths and limitations

Our study sample did not include those who had explicitly left jobs as R-NMAHPs at the time of interview, although some interviewees had moved away from working predominantly in research delivery to become more independent researchers. As such, the experiences of NMAHPs who have left research jobs have not been included in the study, although these would likely make an interesting addition and help elucidate whether the topics we discuss (such as the challenges of boundary spanning and a changed sense of professional identity) might contribute to their decision to leave in addition to other factors, as identified by Boulton and Beer [3].

The interviews for this study were carried out between December 2017 and July 2018. Since then, a number of NIHR-driven initiates have supported the professionalization of the CRP workforce, such as the development of the CRP Directory. Whilst this wider research delivery context has changed since the interviews took place, there were glimmers of recognition of CRPs (albeit usually referred to in other terms, such as “non-nurses”) as an emerging threat or complication to the ongoing establishment and progression of the R-NMAHP workforce. Our analysis and discussion embeds the data in this wider landscape, and highlights a need for further research. Whilst including CRPs in the sample could have provided an interesting dataset to explore alongside the experiences of R-NMAHPs, we suggest that willingness of R-NMAHPs to participate in the study could have been compromised if our recruitment approach had implied a lack of awareness about (or disregard towards) these differences, which previous research demonstrates as meaningful (e.g. [17]). In addition, it would have further complicated an already highly heterogeneous sample. A mitigation of this for future research might be to undertake a separate study on CRPs, analysed concurrently with data on R-NMAHPs.

However, our data are rich and detailed, providing for an in-depth exploration of topics affecting R-NMAHP experiences. In addition, whilst previous studies have typically focused on more specific groupings (such as research nurses only), a strength of our study is the diversity of the sample; for example, our inclusion of nurses, midwives and AHPs has presented opportunities to consider similarities and differences in experiences between the groups. In some cases, participants themselves suggested differences might be based on HCP professional norms and values (such as around uniforms) or factors such as how long research delivery roles had been established in their respective professions. It also allowed for profession-specific expertise and skills utilized in research delivery to be considered, as well as those held across professions (such as good communication skills with patients), when reflecting on the question of the value of NMAHP backgrounds to the role.

## Conclusions

Moving into a research role can mark a significant shift in working practices and responsibilities for nurses, midwives and allied health professionals. Part of this adjustment involves this cadre recognizing that how others perceive them may have changed quite dramatically, impacting how they perceive themselves. This sense of changed identity can draw on real, anticipated and imagined interactions and attitudes of clinical colleagues and patients, as well as the general public whose archetype of “the nurse”, for example, may look very different to the work of a research nurse. For some R-NMAHPs, a move into research may also elicit their own previously held negative assumptions about the role and the motivations of those who occupy the posts. They may have a sense of insecurity or feel that they have “lost” something or diminished their professional identity in some way by moving into research delivery. At the same time, these research roles are a vital part of the infrastructure supporting health research, and R-NMAHPs often become advocates for the value of these endeavours to yield patient benefits. A sense of both loss and pride regarding their roles highlights how R-NMAHPs occupy a difficult space in terms of knowing and articulating their value. Uniforms, name badges and titles are co-constructing markers of professional identity, and the tensions articulated about these highlight the difficulties they face individually and collectively in bridging both the “research” (delivery) aspects and the “professional” (caring) aspects of their roles.

As boundary spanners, R-NMAHP roles connect within and between important groups in research activity. This is an essential part of the job, and their familiarity with clinical working is often an asset; for individuals, however, it can provoke anxiety and a sense of not completely “fitting in”. Comments from others questioning whether R-NMAHPs are “still” nurses, midwives or AHPs can undermine how individuals in the roles might prefer to frame their professional identity, relegating it to the periphery. This finding resonates with work by Gill [13], demonstrating simultaneously how, for some professionals, high levels of commitment and passion about work activities can sit alongside status anxiety. In addition to feeling rejected from their (non-research) clinical colleagues, R-NMAHPs can struggle to affiliate with and expand the “research” aspect of their new titles and responsibilities. Some expressed frustration at career progression and, highlighting confusion regarding research delivery and independent researcher roles, the expectation that they would or could only deliver research, with limited involvement in the design, analysis and dissemination of the studies—components which typically fall outside of the research delivery remit. Some of the individuals we spoke to held independent researcher status, having made a move from “R-NMAHPs” (delivering research) to NMAHPs (involved in all stages of research), but often found the career pathways for progressing this were limited or nonexistent. Also reflected in our data, Boulton and Beer [3] discuss factors affecting the retention of research nurses, with applicability to research midwives and AHPs, such as concern about loss of clinical skills/not learning new ones, the sense that research and researchers are not valued, and the lack of career stability (particularly around short-term contracts) and opportunities for career progression.

Our analysis of R-NMAHP professional identities highlights how individuals themselves try to negotiate, reconcile and embody an answer that pulls components of “research” (delivery) and “professional” (caring) NMAHP roles together. The vast array of backgrounds, skills and knowledge of R-NMAHPs, and the diversity of studies they work on, mean that the value they bring is highly nuanced. However, the landscape of health research means there is also a need to articulate this on a collective scale to secure and advance R-NMAHP careers, particularly in light of NIHR-driven efforts to professionalize CRPs in the United Kingdom. By unpicking the complex boundary-spanning role as it translates into a professional identity, the necessity of existing initiatives to support R-NMAHPs are reiterated, and there are opportunities to identify additional ways to better support them while acknowledging the ever-changing landscape of health research in which they operate. This includes not only support for R-NMAHPs as they undergo adjustment into the role but further opportunities for professionalization, training and developing career pathways alongside (and not at the expense of) other research delivery workforces such as CRPs.

## Data Availability

The datasets used and/or analysed during the current study are available from the corresponding author on reasonable request.

## Abbreviations

AHP: Allied health professional
CRP: Clinical research practitioner
CTO: Clinical trials officer
FDA: Food and Drug Administration
NIHR: National Institute for Health Research
NHS: National Health Service
NMAHP: Nurse, midwife and allied health professional
R-N: Research nurse
R-NMAHP: Research nurse, midwife and allied health professional
R-M: Research midwife
R-AHP: Research allied health professional

## Footnotes

1 Throughout the paper, we refer to “clinical” activities to mean patient care (not only medicine); therefore “clinical colleagues” refers to nurses, midwives and allied health professionals as well as doctors.

2 The professional backgrounds of participants are denoted with quotes: research nurse (R-N), research midwife (R-M) and research allied health professional (R-AHP).

3 A number of NIHR webpages (2019a, b, c, d) about R-NMAHPs cited in this paper no longer exist. Some web pages (NIHR 2019c, d) redirect to a web page on career development in health research more broadly, with the CRP Directory a prominent link. The NIHR web pages (2019a, b) which hosted two strategies, on research nurses and research AHPs respectively, no longer exist and do not appear to have been archived on the NIHR website. We suggest this denotes an approach by the NIHR to amalgamate research delivery staff groups that they formerly distinguished between, and which our paper distinguishes between, which will doubtlessly have implications for the professional identities of those involved.

## References

[1] Allen D (2015). The invisible work of nurses: hospitals, organisations and healthcare.

[2] Allen D (1997). The nursing-medical boundary: a negotiated order. Socio Health Illn.

[3] Boulton M, Beer S (2018). Factors affecting recruitment and retention of nurses who deliver clinical research: a qualitative study. Nurs Open.

[4] Boulton M, Hopewell N (2017). The workforce delivering translational and applied health research: A cross sectional survey of their characteristics, studies and responsibilities. Collegian.

[5] Calnan M, Collyer F (2015). Eliot Freidson: sociological narratives of professionalism and modern medicine. Palgrave handbook of social theory in health, illness and medicine.

[6] Crosno J, Rinaldo S, Black H, Kelley S (2009). Half full or half empty: the role of optimism in boundary-spanning positions. J Serv Res.

[7] Coburn D, Willis E, Albrecht G, Fitzpatrick R, Scrimshaw S (2000). The medical profession: knowledge, power and autonomy. Handbook of social studies in health and medicine.

[8] Coyne I (1997). Sampling in qualitative research. Purposeful and theoretical sampling; merging or clear boundaries?. J Adv Nurs..

[9] Faulkner-Gurstein R, Jones H, McKevitt C (2019). “Like a nurse but not a nurse”: Clinical Research Practitioners and the evolution of the clinical research delivery workforce in the NHS. Health Res Policy and Syst.

[10] Fournier V, Malin N (2000). Boundary work and the ‘unmaking of the professions’. Professionalism, boundaries and the workplace.

[11] Freidson E (1984). The changing nature of professional control. Annu Rev Sociol.

[12] Gieryn T (1983). Boundary-work and the demarcation of science from non-science: strains and interests in professional ideologies of scientists. Am Sociol Rev.

[13] Gill M (2015). Elite identity and status anxiety: an interpretative phenomenological analysis of management consultants. Organization.

[14] Grant S, Guthrie B (2018). Between demarcation and discretion: the medical-administrative boundary as a locus of safety in high-volume organisational routines. Soc Sci Med.

[15] Healthtalk.org. Nurses, midwifes and allied health professionals in research. 2019. https://healthtalk.org/experiences-nurses-midwives-allied-health-professionals-research/overview. Accessed 5 Feb 2020.

[16] Johnson T (1972). Professions and power.

[17] Jones C, Hastings C, Wilson L (2015). Research nurse manager perceptions about research activities performed by non-nurse clinical research coordinators. Nurs Outlook.

[18] McDonald L, Collyer F (2015). Florence nightingale: a research-based approach to health, healthcare and hospital safety. Palgrave handbook of social theory in health, illness and medicine.

[19] Nasir L, Robert G, Fischer M, Norman I, Murrels T, Schofield P (2013). Facilitating knowledge exchange between health-care sectors, organisations and professions: a longitudinal mixed-methods study of boundary-spanning processes and their impact on health-care quality. HS&DR.

[20] NIHR. NIHR Clinical Research Network Research Nursing Strategy 2017–2020. 2018a. https://www.nihr.ac.uk/our-faculty/clinical-research-staff/clinical-research-nurses/Clinical%20Research%20Nurse%20Strategy%202017_2020FINAL.pdf. Accessed 9 Feb 2018.

[21] NIHR. NIHR Clinical Research Network: Developing our Clinical Research Nursing Strategy 2017–2020. 2019a. https://www.nihr.ac.uk/documents/developing-our-clinical-research-nursing-strategy/11500?pr=. Accessed 30 July 2019.

[22] NIHR. NIHR CRN Allied Health Professionals Strategy 2018–2020. 2018b. https://www.nihr.ac.uk/our-faculty/clinical-research-staff/Allied%20Health%20Professionals/Allied%20Health%20Professionals%20Strategy%202018_20.pdf. Accessed 9 Feb 2018.

[23] NIHR. NIHR Clinical Research Network: NIHR CRN Allied Health Professionals 2018–2020. 2019b. https://www.nihr.ac.uk/documents/nihr-crn-allied-health-professionals-strategy-2018-2020/11530. Accessed 30 Jul 2019.

[24] NIHR. Clinical Research Nurses. 2019c. https://www.nihr.ac.uk/our-research-community/clinical-research-staff/clinical-research-nurses/. Accessed 14 May 2020.

[25] NIHR. Allied Health Professionals. 2019d. https://www.nihr.ac.uk/our-research-community/clinical-research-staff/allied-health-professionals.htm. Accessed 14 May 2020.

[26] NIHR. Clinical Research Practitioner (CRP) Directory. 2020. https://www.nihr.ac.uk/health-and-care-professionals/career-development/crp-directory.htm. Accessed 12 Feb 2020.

[27] Powell A, Davies H (2012). The struggle to improve patient care in the face of professional boundaries. Soc Sci Med.

[28] Spilsbury K, Petherick E, Cullum N, Nelson A, Nixon J, Mason S (2008). The role and potential contribution of clinical research nurses to clinical trials. J Clin Nurs.

[29] Timmons S, East L (2011). Uniforms, status and professional boundaries in hospital. Socio Health Illn.

[30] Tinkler L, Smith V, Yiannakou Y, Robinson L (2018). Professional identity and the clinical research nurse: a qualitative study exploring issues having an impact on participant recruitment in research. J Clin Nurs.

[31] Tushman M (1974). Organizational change: an exploratory study and case history.

